# A standardized model of brain death, donor treatment, and lung transplantation for studies on organ preservation and reconditioning

**DOI:** 10.1186/2197-425X-2-12

**Published:** 2014-06-10

**Authors:** Franco Valenza, Silvia Coppola, Sara Froio, Giulia Maria Ruggeri, Jacopo Fumagalli, Alessandro Maria Villa, Lorenzo Rosso, Paolo Mendogni, Grazia Conte, Caterina Lonati, Andrea Carlin, Patrizia Leonardi, Stefano Gatti, Nino Stocchetti, Luciano Gattinoni

**Affiliations:** Dipartimento di Anestesia Rianimazione (Intensiva e Subintensiva) e Terapia del dolore, Fondazione IRCCS Ca’ Granda-Ospedale Maggiore Policlinico, Milan, 20122 Italy; Dipartimento di Fisiopatologia Medico-Chirurgica e dei Trapianti, Università degli Studi di Milano, Milan, 20122 Italy; Unità Operativa di Chirurgia Toracica, Fondazione IRCCS Ca’ Granda-Ospedale Maggiore Policlinico, Milan, 20122 Italy; Unità Operatica Trapianti di Fegato, Fondazione IRCCS Ca’ Granda-Ospedale Maggiore Policlinico, Milan, 20122 Italy; Centro di Ricerche Chirurgiche Precliniche, Fondazione IRCCS Ca’ Granda-Ospedale Maggiore Policlinico, Università degli Studi di Milano, Milan, 20122 Italy

**Keywords:** Brain death, Tissue and organ procurement, Lung transplantation, Organ preservation/methods, Reperfusion injury, Ventilator-induced lung injury

## Abstract

**Background:**

We set a model of brain death, donor management, and lung transplantation for studies on lung preservation and reconditioning before transplantation.

**Methods:**

Ten pigs (39.7 ± 5.9 Kg) were investigated. Five animals underwent brain death and were treated as organ donors; the lungs were then procured and cold stored (Ischemia). Five recipients underwent left lung transplantation and post-reperfusion follow-up (Graft). Cardiorespiratory and metabolic parameters were collected. Lung gene expression of cytokines (tumor necrosis factor alpha (TNFα), interleukin-1 beta (IL-1β), interleukin-6 (IL-6), interferon gamma (IFNγ), high mobility group box-1 (HMGB-1)), chemokines (chemokine CC motif ligand-2 (CCL2-MCP-1), chemokine CXC motif ligand-10 (CXCL-10), interleukin-8 (IL-8)), and endothelial activation markers (endothelin-1 (EDN-1), intercellular adhesion molecule-1 (ICAM-1), vascular cell adhesion molecule-1 (VCAM-1), selectin-E (SELE)) was assessed by real-time polymerase chain reaction (PCR).

**Results:**

Tachycardia and hypertension occurred during brain death induction; cardiac output rose, systemic vascular resistance dropped (*P* < 0.05), and diabetes insipidus occurred. Lung-protective ventilation strategy was applied: 9 h after brain death induction, PaO_2_ was 192 ± 12 mmHg at positive end-expiratory pressure (PEEP) 8.0 ± 1.8 cmH_2_O and FiO_2_ of 40%; wet-to-dry ratio (W/D) was 5.8 ± 0.5, and extravascular lung water (EVLW) was 359 ± 80 mL. Procured lungs were cold-stored for 471 ± 24 min (Ischemia) at the end of which W/D was 6.1 ± 0.9. Left lungs were transplanted and reperfused (warm ischemia 98 ± 14 min). Six hours after controlled reperfusion, PaO_2_ was 192 ± 23 mmHg (PEEP 8.7 ± 1.5 cmH_2_O, FiO_2_ 40%), W/D was 5.6 ± 0.4, and EVLW was 366 ± 117 mL. Levels of IL-8 rose at the end of donor management (BD, *P* < 0.05); CCL2-MCP-1, IL-8, HMGB-1, and SELE were significantly altered after reperfusion (Graft, *P* < 0.05).

**Conclusions:**

We have set a standardized, reproducible pig model resembling the entire process of organ donation that may be used as a platform to test *in vivo* and *ex vivo* strategies of donor lung optimization before transplantation.

**Electronic supplementary material:**

The online version of this article (doi:10.1186/2197-425X-2-12) contains supplementary material, which is available to authorized users.

## Background

Over several decades, lung transplantation has become a consolidated treatment modality. However, the disproportion between organ supply and demand has not been solved. Consequently, donor criteria have been progressively expanded to increase the donor pool [1, 2]. In addition, new techniques such as extracorporeal lung reconditioning [3–5] that allow pharmacological [6, 7], gene [8], or cell [9] therapy have been proposed. As a consequence of these novel opportunities, the concept of organ ‘acceptability’ has been reconsidered: organs that were previously considered unsuitable for transplantation are now well accepted [4, 10–14]. However, while this extended suitability aids solving the organ shortage problem, it also raises several issues, among which the need to implement protocols of treatment in order to preserve over the donation process, if not ameliorate, the function of organs that a few years ago would not have been considered for transplantation.

The aim of this investigation was to set and characterize a pig model that closely resembles the entire process of lung donation and transplantation. Each phase of the clinical procedure, including the pathophysiological changes induced by brain death, the complexity of donor management, and lung transplantation has been carefully reproduced. Further, we have applied a lung-protective ventilatory strategy in the donor animals and during the reperfusion phase of transplantation and measured gene expression of important cytokines, chemokines and markers of endothelial activation throughout the protocol. Hereafter, we present and discuss the results of our investigation.

## Methods

This experimental study was performed after the Ethics Committee of the Fondazione IRCCS Ca’ Granda - Ospedale Maggiore Policlinico and the Italian Ministry of Health approved the protocol (Permit Number: 05/12). All surgeries were performed under anesthesia, and all efforts were made to minimize suffering. Experiments were performed in conformity to the revised Institute of Laboratory Animal Resources, Commission on Life Sciences, National Research Council ‘Guide for the Care and Use of Laboratory Animals’ National Academy Press, Washington, D.C., 1996 (http://www.nap.edu/catalog/5140.html).

**Figure 1:**
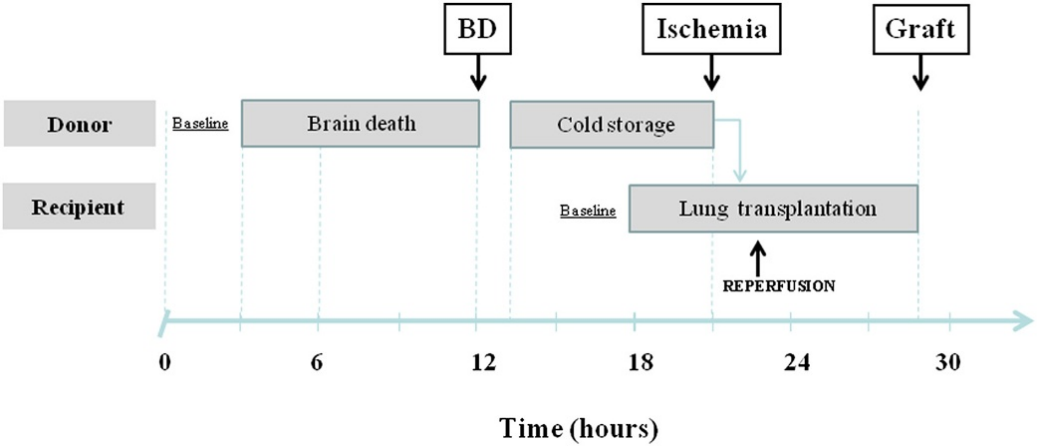
**Schematic overview of the experiment flow.** Each experiment was run using two animals (lung donor and transplantation recipient). The donor underwent induction of brain death followed by organ donor management for a total of 9 h after brain death induction (BD); the lungs were then harvested and cold-stored for 8 h (Ischemia). The recipient pig underwent pneumonectomy and transplantation of the left donor lung, post-reperfusion follow-up was carried on for the following 6 hours (Graft). Arrows in the figure represent the following timings: BD refers to the end of donor management; Ischemia indicates the end of 8 h of cold storage and Graft the end of 6 h follow-up after graft reperfusion.

A schematic overview of the protocol is shown in Figure 1. Each experiment was run using two animals (lung donor and transplantation recipient). The donor underwent induction of brain death followed by organ donor management for a total of 9 h after brain death induction; the lungs were then harvested and cold-stored for 8 h. The recipient pig underwent pneumonectomy and transplantation of the left donor lung; post-reperfusion follow-up was carried on for the next 6 h.

### Anesthesia and monitoring

Details of anesthesia and monitoring are described in Additional file 1. Briefly, animals received an intramuscular injection of olanzapine and tiletamine 2 mg (Zoletil, VIRBAC s.r.l., Milan, Italy) and medetomidine 1 mg (Domitor, Pfizer Animal Health, Exton, PA, USA and Div. of Pfizer Inc., New York, NY, USA). A continuous intravenous infusion of propofol (Diprivan, AstraZeneca, Basiglio, Milan, Italy) 10 to 15 mg/Kg/h and medetomidine 3 to 6 μg/Kg/h was then started. A number of catheters were positioned and secured in place to measure arterial, central venous, and pulmonary artery pressure throughout brain death induction, donor treatment, recipient surgery, and follow-up. Cardiac output was measured by the Swan-Ganz catheter. Extravascular lung water (EVLW), global end diastolic volume (GEDV), and stroke volume variation (SVV) were monitored by PiCCO2® (PULSION Medical Systems, AG, Stahlgruberring 28, München, Deutschland, Germany) monitor. Analysis of pO_2_, pCO_2_, pH, and derived variables (base excess, HCO_3_), together with electrolytes (Na^+^, K^+^, Ca^2+^, Cl^−^), glucose, and lactate concentrations was performed on arterial and central venous samples (Radiometer ABL 800 Flex, Radiometer Medical ApS, Brønshøj, Denmark). Urinary electrolytes concentrations were measured by K.I.N.G.® (Orvim s.r.l., Paderno Dugnano, Milan, Italy). Blood chemistry (blood urea nitrogen (BUN), creatinine, serum glutamic oxaloacetic transaminase (SGOT), serum glutamic-pyruvic transaminase (SGPT), troponin T) was also assessed at baseline and at the end of donor management (BD).

### Induction of brain death

To induce brain death, a slight modification of the protocol described by Purins et al. was used [15]. Briefly, to rise intracranial pressure (ICP), an 18-Fr Foley catheter (Willy Rush AG, Kernen, Germany) was placed in the epidural space and progressively inflated with saline (1.5 mL every 10 min). ICP was continuously measured by connecting a subdural probe (Integra Neurosciences, TraumaCath, Enterprise Drive, Plainsboro, NJ, USA) to a pressure transducer (TruWave, Edwards Lifesciences LLC, Irvine, CA, USA). The balloon of the Foley catheter was inflated until cerebral perfusion pressure, calculated as mean arterial pressure (MAP) minus ICP (CPP = MAP − ICP), was less than 0 mmHg. At each step of inflation, the microballoon of a 5-Fr pulmonary arterial catheter (Edwards Lifesciences LLC) positioned intraparenchymally was inflated with 1 mL of air; the maneuver was used to calculate intracranial compliance (IC = 1/ΔICP). Once CPP was negative, muscle paralysis, anesthesia, and analgesia were discontinued. Brain death was confirmed at the end of 60 min of CPP < 0 mmHg and before lung retrieval. Clinical signs of brain death included the absence of corneal reflex and the absence of coughing in response to tracheal suctioning. An apnea test was also performed. This was conducted during continuous positive airway pressure verifying the absence of breathing, confirmed by the absence of esophageal pressure deflections (SmartCath Viasys, Palm Springs, CA, USA) while pCO_2_ was above 60 mmHg (verified by arterial blood gas analysis). In three animals, brain death was also confirmed by electroencephalography (B. E. Light, EBNeuro S.p.A., Florence, Italy) [16]. In these animals, monitoring was extended throughout the protocol procedure, from before brain death induction to the end of brain death donor management.

**Figure 2:**
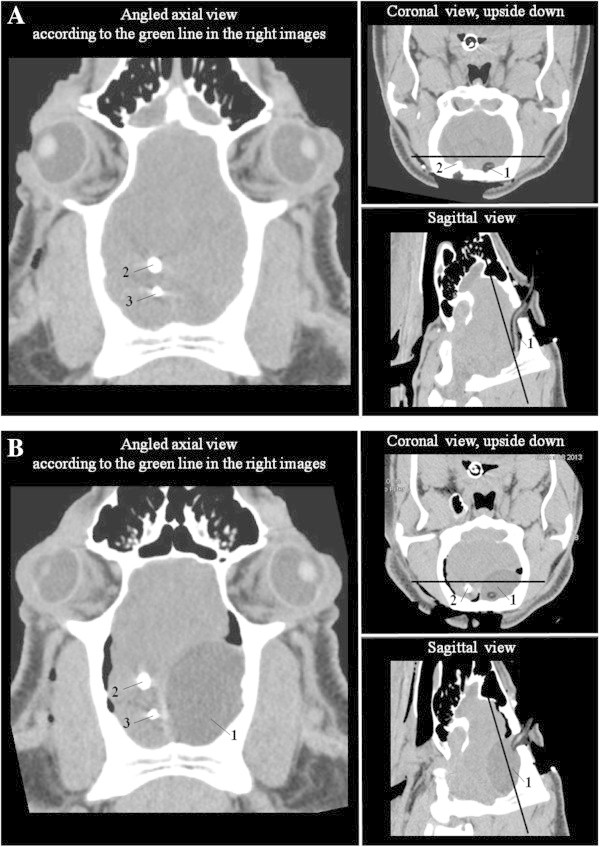
**CT scan analysis of the skull at baseline (A) and after brain death induction (B) in one exemplificative animal.** 1, epidural Foley catheter; 2, ICP monitoring catheter; 3, intraparenchymal Swan-Ganz catheter for measuring intracranial compliance.

An exemplificative CT scan shows the position of the intracranial catheters and the cerebral parenchyma deformation before and after the inflation of the epidural Foley catheter (Figure 2). A representative diagram of the site of brain catheter placement (Additional file 2: Figure S1), a radiograph of the epidural Foley catheter once inflated in the pig cranium (Additional file 3: Figure S2), and a photograph showing brain to inflated balloon proportions (Additional file 4: Figure S3) can be found in the Additional files.

### Organ donor management

After brain death was confirmed, donor animals were treated according to standard physiological targets for the next 6 h [17, 18]. Cardiovascular targets included MAP > 60 mmHg, central venous pressure (CVP) between 5 and 8 mmHg and urine output > 1.5 mL/Kg/h. Hypotension was treated with vasoactive drugs when hemodynamic instability persisted despite adequate volume resuscitation. Norepinephrine was the first choice drug when systemic vascular resistances were below 800 dyn · s · cm^−5^. 1-Desamino-8-D-arginine vasopressin (0.125 to 0.250 μg endovenous) was given when diabetes insipidus occurred, defined as urine output > 4 mL/Kg/h, urinary specific gravity < 1.005, and blood osmolarity > 300 mOsm/Kg, calculated as [2 × Na] + [glucose/18]. We decided to use methylprednisolone by protocol (15 mg/kg) but not thyroid hormones as their use is suggested only when hemodynamic instability persists despite aggressive treatment [19–22]. After brain death confirmation, mechanical ventilation was set according to a lung-protective ventilatory strategy [18, 23]: tidal volumes of 6 to 8 mL/Kg, positive end-expiratory pressure (PEEP) of 8 to 10 cmH_2_O, and respiratory rate set to maintain pCO_2_ lower than 50 mmHg with 7.35 < pH < 7.45. Recruitment maneuvers were performed at the end of each apnea test allowing ten consecutive breaths with an inspiratory pressure target of 40 cmH_2_O above PEEP of 5 cmH_2_O.

### Lung harvest and cold storage

A median sternotomy was performed, the thymus removed, the pleura carefully dissected, and the pericardium opened. The superior and inferior cava veins were encircled with silk ties and a bolus of 20,000 U heparin (Pharepa, Pharmatex Italia s.r.l., Milan, Italy) was injected into the jugular vein. Five minutes after the heparin bolus, a cannula was inserted into the main pulmonary artery. A bolus of 250 μg of alprostadil (Prostin, Pfizer Manufacturing Belgium N.V., Puurs, Belgium) was then injected into the main pulmonary artery. The superior and inferior cava veins were then ligated, the ascending aorta clamped, and the left atrial appendage transected. The lungs were then flushed with 60 ml/Kg of cold Perfadex® (Vitrolife Sweden Instruments AB, Billdal, Sweden) at a height of 30 cm above the heart. During the perfusion with the preservation solution, respiratory rate was decreased and FiO_2_ increased to 100%. Ventilation was discontinued when the heart-lung block was removed. Just before removing the lungs, the trachea was clamped with lungs fully inflated. After removal from the thoracic cavity, the heart-lung block was placed on ice, the heart removed and the lung perfused with Perfadex® in a retrograde manner (i.e., from the left veins to the pulmonary artery). Thereafter, the lungs were placed in a plastic bag (Vitrolife) containing Perfadex® solution and stored on ice for 8 h while continuously monitoring lung surface temperature (Medical Temperature Probes, Siemens S.p.A., Milan, Italy).

### Lung transplantation

A detailed description of the surgical procedure of lung transplantation is reported in Additional file 1. Briefly, the recipient pig was placed in the right lateral decubitus position, a left thoracotomy performed just below the tip of the scapula, and a left pneumonectomy completed. The donor lung was prepared on the back table while on ice then transferred to the thoracic cavity, and bronchial, pulmonary artery, and venous anastomoses were performed. The atrial clamp was then released to de-air the donor lung in a retrograde manner. Thereafter, the artery clamp was opened step by step in 10 min; the bronchial clamp was then removed allowing left lung ventilation.

Ventilation protocol was as follows: during pneumonectomy tidal volume was set at 6 to 8 mL/Kg, PEEP at 5 cmH_2_O, and FiO_2_ at 40%. By protocol, increments of PEEP were allowed if PaO_2_ was <100 mmHg or SpO_2_ < 95%. After reperfusion, pressure-controlled mode was instituted maintaining the same target volume of 6 to 8 mL/Kg; PEEP was set at 8 cmH_2_O, and a recruitment maneuver was performed (total target pressure of 45 cmH_2_O) 45 min after the start of controlled reperfusion. During post-reperfusion follow-up, if SpO_2_ was <90%, increments of PEEP were allowed up to 15 cmH_2_O; thereafter, FiO_2_ had to be increased in case of persistent hypoxia. Respiratory rate was set to maintain pCO_2_ below 70 mmHg and/or pH > 7.25. In case of persistent hypercapnia, increases of tidal volume were allowed. Cardiovascular targets included MAP > 60 mmHg, CVP between 5 and 8 mmHg, and urine output > 1.5 mL/Kg/h; care was given to administer the least amount of fluid possible. Cardiovascular, respiratory, and metabolic parameters were collected throughout reperfusion and post-reperfusion follow-up.

### Assessment of lung function and gene expression

Oxygenation was assessed measuring partial pressure of oxygen from peripheral arterial blood samples (PaO_2_) and calculating the oxygenation index (OI) as (FiO_2_ × Paw_m_)/PaO_2_, where Paw_m_ is mean airway pressure. The physiologic dead space fraction (VD/VT) was computed according to the following formula: VD/VT = (PaCO_2_ − PECO_2_)/PaCO_2_, where PECO_2_ is the mixed expired carbon dioxide partial pressure obtained by means of expiratory air sampling [24]. EtCO_2_/PaCO_2_ was also calculated. Respiratory mechanics was assessed partitioning lung and chest wall components by means of an esophageal balloon catheter, as detailed in the Additional file 1. End-expiratory lung volume (EELV) was measured using the closed circuit helium technique [25]. As index of lung edema, EVLW and wet-to-dry lung ratio (W/D) were measured according to standard procedures (see Additional file 1). Transcriptional expression of tumor necrosis factor alpha (TNFα), interleukin-1 beta (IL-1β), interleukin-6 (IL-6), interferon gamma (IFNγ), high mobility group box-1 (HMGB-1), chemokine CC motif ligand-2 (CCL2-MCP-1), chemokine CXC motif ligand-10 (CXCL-10), interleukin-8 (IL-8), endothelin-1 (EDN-1), intercellular adhesion molecule-1 (ICAM-1), vascular cell adhesion molecule-1 (VCAM-1), and selectin-E (SELE) was evaluated by real-time reverse transcription polymerase chain reaction (PCR) analysis performed on total mRNA isolated from lung tissue samples [26, 27]. A detailed description of the technique of mRNA isolation and gene expression measurement may be found in the Additional file 1.

### Statistical analysis

All results are presented as mean ± standard deviation (SD), unless otherwise specified. Continuous variables referring to neurological, cardiovascular, respiratory, and metabolic function were analyzed within donor or recipient animals by ANOVA for repeated measures followed, were appropriate, by Bonferroni test for all pairwise multiple comparisons. Data that were not normally distributed were investigated by ANOVA on ranks followed by Dunn's test for all pairwise comparisons. Parameters of lung function (PaO_2_/FiO_2_, OI, VD/VT, EELV, EVLW, EtCO_2_/PaCO_2_) taken before (baseline) and after BD and after lung transplantation (Graft) were assessed by means of ANOVA. The gene expression of lung biomarkers and W/D ratio were investigated at the end of BD, after 8 h of cold storage (Ischemia) and at the end of reperfusion follow-up (Graft) by one-way ANOVA and compared to controls (Control). For this purpose, three sham operated pigs were considered. *P* < 0.05 was accepted as significant. Data were analyzed using Sigma Plot version 11.0 (Systat Software, Inc., GmbH, Munich, Germany).

## Results

A total of ten domestic pigs (five donors and five recipients) were consecutively included in the study and investigated as described in Figure 1. Three additional sham-operated animals were included and their lungs used as controls.

### Induction of brain death and donor management

**Figure 3:**
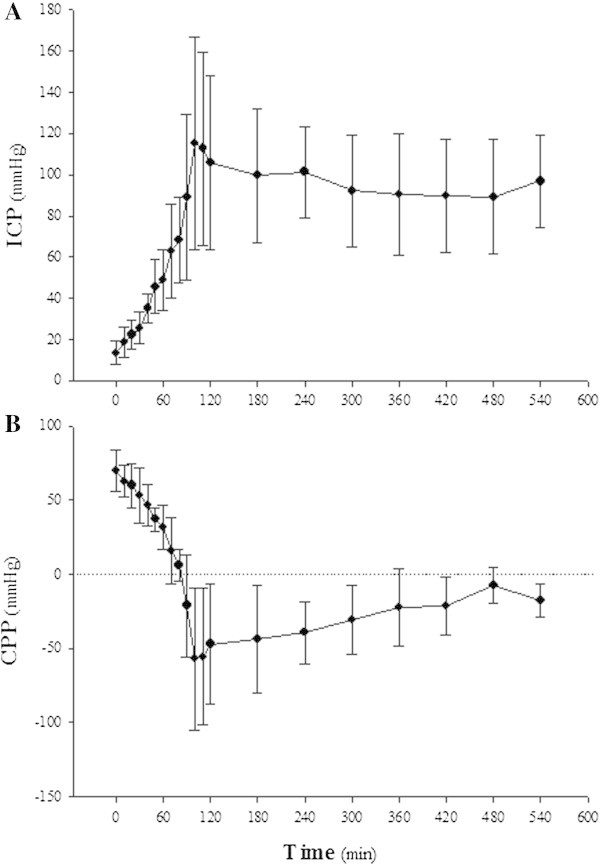
**Intracranial pressure (ICP) and cerebral perfusion pressure (CPP).** The rise of ICP **(A)** and the decrease of CPP **(B)** during the induction of brain death and during donor management are shown in the figure. The error bars show the standard deviation of the mean.

**Figure 4:**
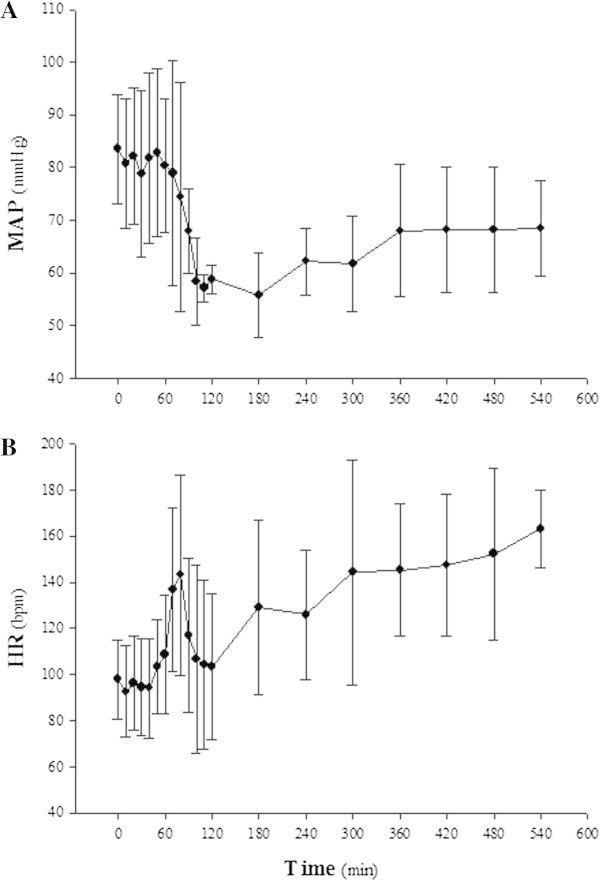
**Hemodynamic response during the induction of brain death and during the following hours of donor management. (A)** Mean arterial pressure (MAP). **(B)** Heart rate (HR). The error bars show the standard deviation of the mean.

As shown in Figure 2, the inflation of the Foley catheter caused sovratentorial mass expansion that caused an increase in ICP (Figure 3A) and a decrease in CPP (Figure 3B). Intracranial compliance significantly dropped from 0.24 ± 0.13 to 0.02 ± 0.01 mL/mmHg (*P* < 0.05). When CPP was close to zero, transient hypertension and sustained tachycardia occurred, followed by severe hypotension (Figure 4). In all donor animals, clinical signs confirmed brain death at the end of brain death induction and throughout donor management. A representative pattern of electroencephalogic activity before and after brain death induction is shown in Additional file 5: Figure S4.

Cardiovascular, respiratory, and metabolic parameters collected during the hours of donor management are shown in Table 1. Over time, cardiac output significantly rose (*P* < 0.05) and (*P* < 0.05). Volume load (average 8 ± 4 mL/Kg/h) and noradrenaline infusion (average 0.04 ± 0.02 μg/Kg/min) were necessary in all animals. Central venous pressure slightly rose over time (*P* < 0.05); stroke volume variation was always within normal ranges. Lactate rose by the end of donor management (*P* < 0.05). Atrial fibrillation occurred in three pigs: infusion of amiodarone (150 mg in 250 mL dextrose 5% over a period of 20 to 30 min through a central venous catheter) decreased ventricular rate but did not revert to sinus rhythm. Clear signs of diabetes insipidus occurred in four out of five cases and were treated with 1-desamino-8-D-arginine vasopressin (1 to 3 μg) [28]. The lung-protective ventilatory strategy set after brain death confirmation resulted in a higher PEEP level (*P* < 0.05), lower tidal volume (*P* < 0.05), and higher respiratory rate (*P* < 0.05); PaCO_2_ and pH were not significantly different from baseline, while EtCO_2_ was higher (*P* < 0.05). Insulin administration was necessary to maintain blood concentrations of glucose to levels that were similar to baseline throughout the donor management protocol. Hypernatremia developed over time (*P* < 0.05) and was associated with a lower concentration of sodium in the collected urine (*P* < 0.05).

**Table 1:** **Donor parameters**

	Baseline	Brain death diagnosis	Treatment	Procurement	***P*** value
Temperature, °C	36.3 ± 0.9	37.7 ± 1.3^a^	37.9 ± 1.2^a^	38.2 ± 0.7^a^	<0.05
Heart rate, beats/min	100 ± 20	129 ± 38^a^	145 ± 28^a^	163 ± 17^ab^	<0.05
Mean arterial pressure, mmHg	75 ± 10	56 ± 8	68 ± 13	75 ± 10	<0.05^*^
Pulmonary artery pressure, mmHg	18 ± 3	21 ± 4	19 ± 4	21 ± 4	0.100
Wedge pressure, mmHg	11 ± 3	13 ± 3	14 ± 1	13 ± 2	0.392
Central venous pressure, mmHg	5 ± 3	7 ± 3	7 ± 3	8 ± 3^a^	<0.05
Cardiac output, L/min	2.9 ± 0.2	3.5 ± 0.6	4.4 ± 1.1^a^	5.1 ± 0.8^ab^	<0.05
Systemic vascular resistance, dyn · s · cm^−5^	1,900 ± 269	1,146 ± 187^a^	1,127 ± 156^a^	1,051 ± 177^a^	<0.05
Pulmonary vascular resistance, dyn · s · cm^−5^	195 ± 119	180 ± 65	100 ± 87	134 ± 79	0.103
Stroke volume variation, %	9 ± 2	10 ± 3	12 ± 5	11 ± 4	0.326
Global end diastolic volume, mL	360 ± 53	361 ± 76	394 ± 74	416 ± 108	0.564
Fluid balance, mL	−507 ± 1,249	201 ± 1,360	399 ± 1,396	350 ± 1,707	0.136
Urine output, mL/Kg/h	5.2 ± 1.8	3.1 ± 0.6	8.3 ± 12.5	7.8 ± 5.2	0.521
Lactate, mmol/L	1.7 ± 0.5	2.1 ± 0.8	2.5 ± 2.3	4.8 ± 1.8	<0.05^*^
Oxygen delivery, mL/min	282 ± 31	285 ± 65	411 ± 95	483 ± 113^ab^	<0.05
Respiratory rate, breaths/min	14 ± 3	17 ± 2^a^	19 ± 3^a^	19 ± 3^ab^	<0.05
Tidal volume, mL	328 ± 50	284 ± 30^a^	286 ± 23^a^	288 ± 21^a^	<0.05
Peak airway pressure, cmH_2_O	17 ± 1	19 ± 1	22 ± 2^a^	23 ± 2^ab^	<0.05
Plateau airway pressure, cmH_2_O	12 ± 1	13 ± 1	14 ± 1	14 ± 1	0.061
Mean airway pressure, cmH_2_O	8 ± 0	11 ± 0^a^	12 ± 1^a^	12 ± 2^a^	<0.05
Positive end-expiratory pressure, cmH_2_O	5 ± 0	7 ± 1^a^	8 ± 2^a^	8 ± 2^a^	<0.05
End-tidal CO_2_, mmHg	43.0 ± 7.6	48.5 ± 6.4	48.9 ± 4.6	51.3 ± 2.8^a^	<0.05
PaCO_2_, mmHg	40.5 ± 5.7	45.6 ± 3.2	43.6 ± 2.5	45.6 ± 5.2	0.168
pH	7.429 ± 0.036	7.411 ± 0.046	7.402 ± 0.057	7.358 ± 0.057	0.127
Glucose, g/dL	169 ± 56	147 ± 45	160 ± 82	162 ± 55	0.472
Na^+^, mEq/L	137.0 ± 4.3	137.6 ± 6.1	141.2 ± 4.1	144.2 ± 5.3^ab^	<0.05
K^+^, mEq/L	4.2 ± 0.5	4.5 ± 0.5	4.2 ± 0.3	4.0 ± 0.1	0.375
Ca^++^, mEq/L	1.26 ± 0.01	1.18 ± 0.09	1.19 ± 0.06	1.17 ± 0.04^a^	<0.05
Cl^−^, mEq/L	99.6 ± 2.5	98.6 ± 3.5	101.4 ± 2.9	106.0 ± 5.0^abc^	<0.05
Na^+^_urinary_, mEq/L	64.3 ± 20.4	60.8 ± 31.7	19.7 ± 18.2	7.3 ± 7.3^ab^	<0.05
K^+^_urinary_, mEq/L	29.5 ± 7.3	53.7 ± 23.2	23.2 ± 19.6	6.4 ± 3.3^b^	<0.05
pH_urinary_	6.610 ± 0.320	6.821 ± 0.132	6.741 ± 0.631	6.229 ± 0.512	0.182
Specific gravity_urinary_	1.016 ± 2.5	1.011 ± 2.8	1.005 ± 0.0	1.006 ± 7.6^a^	<0.05

Table 1 shows respiratory, hemodynamic, and metabolic parameters and urinary electrolytes of donor pigs collected at the end of surgery (baseline), after the induction and diagnosis of brain death (brain death diagnosis), 3 h after brain death confirmation (treatment), and at procurement (procurement, 9 h after brain death induction). Data are presented as mean ± standard deviation. One-way ANOVA repeated measures. *P* < 0.05 accepted as significant: ^a^vs. Baseline, ^b^vs. Brain Death, and ^c^vs. 3 h. *P < 0.05: When we tested the differences of main arterial pressure between the time points considered, a statistically significant difference was found (*P* < 0.05). To isolate the group or groups that differ from the others, a multiple comparison procedure was used (Bonferroni *t* test). The statistical software we used (Sigma Stat) is such that when no significant difference is found between the two groups with the higher difference of mean values, a result of ‘Do Not Test’ is provided by Bonferroni *t* test for the all other enclosed comparison. A ‘Do Not Test’ should be treated as if there is no significant difference between the means, even though the ANOVA test indicates that this is the case. This apparent discrepancy is due to the fact that differences are close to significance (*P* = 0.06) but below threshold (set at 0.05). This result may therefore be considered only as a nonsignificant trend.

At the end of donor management, hepatic enzymes were not significantly different from baseline (SGOT 41.4 ± 22 vs. 151.2 ± 130.2 U/L, *P* = 0.145; SGPT 52.9 ± 10.1 vs. 38.2 ± 14.2 U/L, *P* = 0.124); creatinine and BUN were slightly but significantly higher (creatinine 1.1 ± 0.1 vs. 1.5 ± 0.2 mg/dL, *P* < 0.05; BUN 14.2 ± 2.2 vs. 36.5 ± 5.26 mg/dL, *P* < 0.05); there was a trend towards a rise of cardiac troponin T (10.5 ± 10.8 vs. 31.6 ± 15.2 pg/mL, *P* = 0.082).

### Lung harvest, preservation, and transplantation

There were no complications concomitant to lung perfusion, harvest, and back table surgery. Temperature of the graft was always below 8°C during cold preservation. Time from cross clamp to reperfusion was 569 ± 28 min, of which 470 ± 24 min of cold and 98 ± 14 min of warm ischemia, respectively. Surgery was accomplished without major complications. Respiratory and hemodynamic data in the recipient are shown in Table 2; the data referring to the controlled reperfusion are shown in Additional file 6: Table S1 of the supplement material. Positive end-expiratory pressure was increased over time after reperfusion (*P* < 0.05). Mean arterial pressure and cardiac output progressively dropped (both *P* < 0.05). Lactate did not change over time, and no signs of inadequate perfusion were present.

**Table 2:** **Recipient parameters**

	Baseline	Reperfusion	1 h	4 h	6 h	***P*** value
Temperature, °C	37.2 ± 0.8	37.9 ± 0.6	38.0 ± 0.9	37.9 ± 1.2	38.2 ± 1.2	0.153
Heart rate, beats/min	102 ± 24	103 ± 24	102 ± 28	105 ± 19	104 ± 4	0.891
Mean arterial pressure, mmHg	114 ± 19	99 ± 20	91 ± 21	80 ± 6^a^	76 ± 10^a^	<0.05
Pulmonary artery pressure, mmHg	23 ± 5	25 ± 4	25 ± 3	24 ± 4	26 ± 4	0.424
Central venous pressure, mmHg	9 ± 4	6 ± 2	8 ± 3	8 ± 1	9 ± 2	0.105
Cardiac output, L/min	3.6 ± 0.4	4.2 ± 1.2	3.3 ± 0.4	3.3 ± 0.7	3.1 ± 0.6^b^	<0.05
Systemic vascular resistance, dyn · s · cm^−5^	2,433 ± 396	1,877 ± 681	2,101 ± 763	1,787 ± 427	1,766 ± 407	0.183
Stroke volume variation, %	10 ± 4	6 ± 2	6 ± 1	9 ± 5	9 ± 4	0.385
Global end diastolic volume, mL	464 ± 57	436 ± 134	386 ± 51	456 ± 115	414 ± 109	0.890
Urine output, mL · Kg/h	4.3 ± 3.0	3.1 ± 0.7	4.9 ± 3.0	3.6 ± 2.2	2.0 ± 0.7	0.104
Lactate, mmol/L	1.2 ± 0.4	0.7 ± 0.1	1.0 ± 0.6	0.8 ± 0.2	0.8 ± 0.1	0.382
Oxygen delivery, mL/min	473 ± 65	628 ± 199	459 ± 61	428 ± 90^b^	385 ± 81^b^	<0.05
Respiratory rate, breaths/min	18 ± 0	19 ± 4	19 ± 2	17 ± 4	17 ± 4	0.825
Tidal volume, mL	331 ± 47	295 ± 42	321 ± 99	363 ± 136	343 ± 116	0.393
Peak airway pressure, cmH_2_O	17 ± 1	20 ± 5	20 ± 3	21 ± 4	22 ± 5	0.157
Plateau airway pressure, cmH_2_O	11 ± 1	12 ± 2	15 ± 0	17 ± 3^a^	16 ± 4	<0.05
Mean airway pressure, cmH_2_O	8 ± 0	9 ± 1	12 ± 2^ab^	13 ± 2^ab^	12 ± 2^ab^	<0.05
Positive end-expiratory pressure, cmH_2_O	5 ± 0	6 ± 2	8 ± 1^ab^	9 ± 1^ab^	9 ± 2^ab^	<0.05
End-tidal CO_2_, mmHg	47.7 ± 5.2	55.4 ± 7.2	56.6 ± 4.5	49.0 ± 8.7	52.5 ± 6.2	0.096
PaCO_2_, mmHg	46.6 ± 5.2	52.0 ± 5.0	51.6 ± 8.4	41.2 ± 6.7	49.0 ± 11.3	0.053
pH	7.399 ± 0.029	7.349 ± 0.054	7.383 ± 0.057	7.456 ± 0.060^b^	7.403 ± 0.086	<0.05
Glucose, g/dL	161 ± 52	134 ± 70	112 ± 59	119 ± 32	124 ± 24	0.168
Na^+^, mEq/L	138.2 ± 0.8	140.3 ± 4.0	141.8 ± 3.5	140.2 ± 4.6	140.7 ± 4.5	0.226
K^+^, mEq/L	3.8 ± 0.1	4.7 ± 0.3	4.8 ± 0.8^a^	5.3 ± 0.7^a^	5.1 ± 0.7^a^	<0.05
Ca^++^, mEq/L	1.32 ± 0.07	1.20 ± 0.06^b^	1.23 ± 0.07^a^	1.16 ± 0.08^abc^	1.15 ± 0.07^abc^	<0.05
Cl^−^, mEq/L	98.8 ± 3.6	99.3 ± 2.1	101.0 ± 3.5	101.0 ± 3.7	101.0 ± 5.4	0.829
Na^+^_urinary_, mEq/L	49.4 ± 19.9	-	-	60.5 ± 40.3	-	0.475
K^+^_urinary_, mEq/L	38.1 ± 21.4	-	-	95.9 ± 46.8	-	0.053
pH_urinary_	6.801 ± 0.843	-	-	6.702 ± 0.978	-	0.875

Table 2 shows respiratory, hemodynamic, and metabolic parameters and urinary electrolytes of recipient pigs collected after surgery (Baseline), at the beginning of reperfusion (Reperfusion) and after 1, 4, and 6 h after reperfusion. Data are presented as mean ± standard deviation. One-way ANOVA repeated measures. *P* < 0.05 accepted as significant: ^a^vs. Baseline, ^b^vs. Reperfusion, and ^c^vs. 1 h.

### Assessment of lung function and gene expression

Table 3 shows functional respiratory parameters in the donor and recipient animals. The ratio between EtCO_2_ and PaCO_2_, elastance of the respiratory system, EELV and functional dead space were not significantly different at BD or at Graft. There were no signs of lung edema in either BD or Graft, as assessed by EVLW and W/D lung ratio. In three cases, oxygenation of the implanted lung (Graft) was assessed after reperfusion by selective left pulmonary vein and artery blood gas analysis: arterial PaO_2_/FiO_2_ was 532 ± 19 mmHg with PvO_2_ of 34 ± 5 mmHg.

**Table 3:** **Lung function parameters**

	Donor		Recipient	
	Baseline	Before procurement	After reperfusion	***P*** value
PaO_2_/FiO_2,_ mmHg	540 ± 32	480 ± 31	532 ± 19	0.564
Elastance of respiratory system, cmH_2_O/L	20.5 ± 7.9	18.4 ± 7.7	22.6 ± 6.9	0.687
Elastance of lung, cmH_2_O/L	12.9 ± 5.2	15.3 ± 7.7	15.3 ± 7.0	0.846
End expiratory lung volume, mL	735 ± 187	803 ± 312	749 ± 213	0.900
Physiologic dead space fraction	0.54 ± 0.03	0.51 ± 0.07	0.49 ± 0.08	0.631
End-tidal CO_2_/PaCO_2_	1.06 ± 0.04	1.13 ± 0.09	1.19 ± 0.08	0.133
Extravascular lung water, mL	359 ± 79	359 ± 80	366 ± 117	0.949
Wet/dry ratio	6.2 ± 7.0	5.8 ± 0.5	5.6 ± 0.6	0.629

Table 3 shows functional lung parameters in the donor and recipient animals. ‘Before procurement’ refers to data taken at the end the donation process at the time of lung procurement, except for wet-to-dry ratio that was measured in sham-operated animals. *BD* refers to data taken at the end of donor management; ‘After reperfusion’ refers to data taken at the end of post-reperfusion follow-up. Oxygenation of the implanted lung (After reperfusion, *n* = 3) was assessed by selective left pulmonary vein blood gas analysis. Data are presented as mean ± standard deviation. One-way ANOVA. *P* < 0.05 accepted as significant.

**Figure 5:**
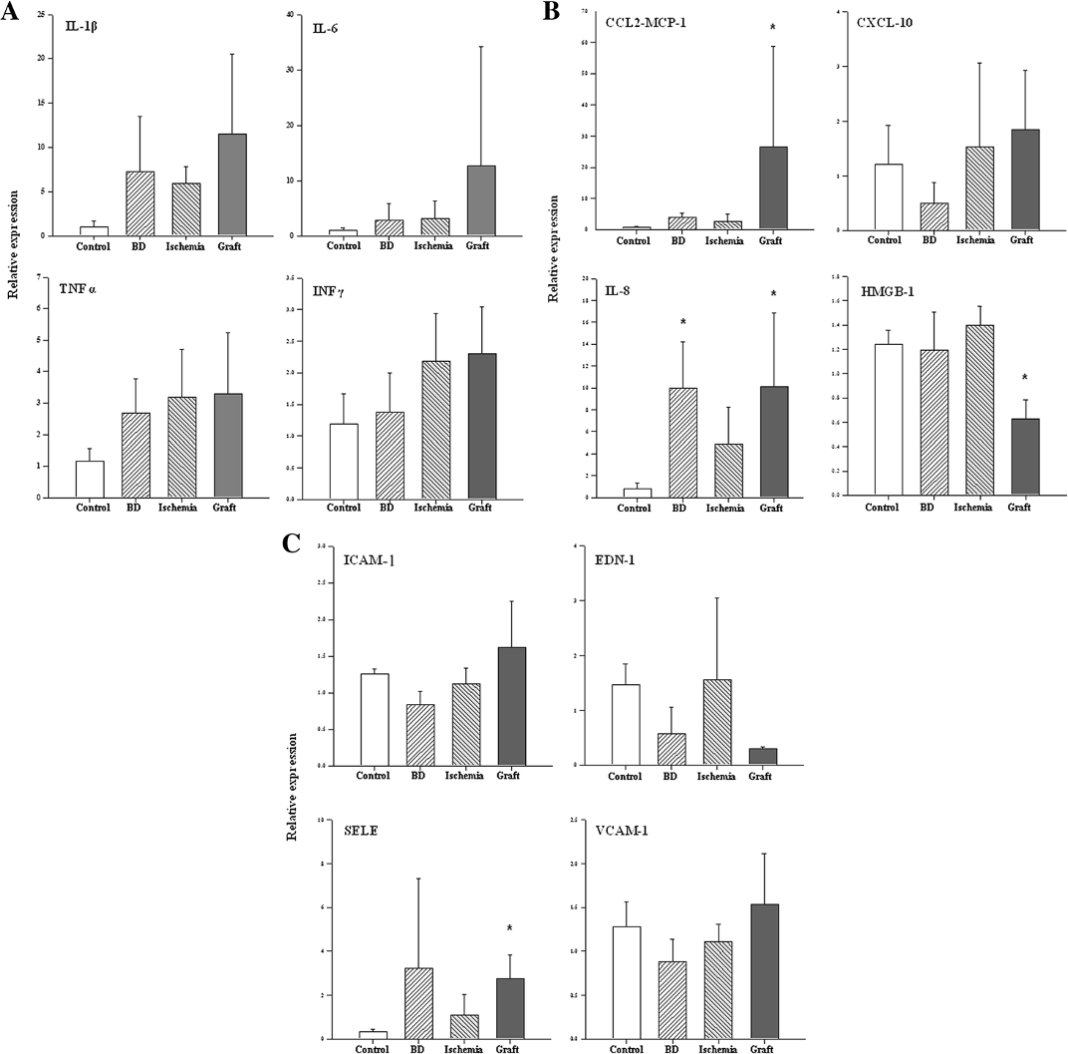
**Gene expression of lung inflammatory mediators obtained by real-time PCR in lung homogenates.** Lung samples for gene expression analysis were obtained at BD (at the end of donor management), after 8 h of cold storage (Ischemia), at the end of reperfusion follow-up (Graft) and compared to controls (Control). Cytokines, interleukin-1 beta (IL-1β), interleukin-6 (IL-6), tumor necrosis factor alpha (TNF α), and interferon gamma (IFN γ) are shown in **(A)**; chemokines, chemokine C-C motif ligand 2 (CCL2-MCP-1), chemokine CXC motif ligand 10 (CXCL-10), interleukin-8 (IL-8), and the oxidative stress index high mobility group box-1 (HMGB-1) are shown in **(B)**; endothelial mediators: endothelin-1 (EDN-1), intercellular adhesion molecule-1 (ICAM-1), vascular cell adhesion molecule-1 (VCAM-1) and selectin-E (SELE) are shown in **(C)**. The error bars show the standard deviation of the mean. One-way ANOVA vs. Control. *P* < 0.05 accepted as significant: asterisk (*) vs. Control.

Figure 5 shows results of gene expression analysis in lung samples obtained at control, BD, cold ischemia (Ischemia) and after transplantation (Graft). Expression levels of the cytokines TNF α, IL-1β, IL-6, and IFN γ were not significantly different from control expression at any of the examined points (panel A). Increased expression of the chemokines CCL2-MCP-1 at Graft (*P* < 0.05) and IL-8 at both BD and Graft (*P* < 0.05) was observed (panel B). CXCL-10 was not altered at either BD, Ischemia, or Graft, whereas expression of HMGB-1 was significantly lower at Graft (*P* < 0.05, panel B). Adhesion molecules EDN-1, ICAM-1, and VCAM-1 were not affected by either BD, Ischemia, or Graft, while the expression of SELE was significantly higher at Graft (*P* < 0.05, panel C).

## Discussion

This study describes a pig model of brain death, donor management, and lung transplantation that closely resembles clinical conditions. The research effort was to reproduce each critical phase of donation and transplantation in a standardized and optimized fashion and to integrate the clinical approach with biofunctional evidence of lung injury.

Apart from decapitation [29] and cerebral hemorrhage [30], most of the animal investigations on brain death have utilized models of sovratentorial mass expansion to rise ICP [26, 31–36]. While Neyrinck et al. and Ryan et al. induced brain death by an explosive rise of ICP [34, 35], we elected to rise ICP slowly reproducing the protocol recently described by Purins et al. [15]. The model we used resembles that of a progressively expanding mass that brings to complete ischemia. Despite the specific limitations of an animal model, it could resemble an ICP rise that would occur in case of an untreated trauma or a spontaneous cerebral hemorrhage.

Increased intracranial pressure was associated with a decrease in CPP and a drop of intracranial compliance. Similarly to Purins et al., ICP rise was associated with a transient hypertension and tachycardia, followed by severe hypotension. These phenomena occurred at CPP values similar to those recently described in a refinement paper of Purins et al. [33], further confirming the validity of the model. In our model, clinical signs of brain death were evident after 1 h of negative CPP in all animals, including assessment of corneal reflex, coughing in response to tracheal suctioning and execution of an apnea test. Moreover, in a subset of animals, the absence of brain perfusion was confirmed by electroencephalography.

Intracranial pressure and CPP were maintained for an extended number of hours to better reproduce the clinical setting. This is a major distinction relative to previous investigations. Indeed, observation times after brain death were 120 min in Purins' research [15], 300 in Neyrinck [34]; 360 in Lyons [31], McLean [32], and Barklin [26], and 480 in Hvas [30]. We prolonged the time from brain death to lung harvested after a total of 540 min after cerebral mass expansion. Even if this time was not as long as that of the investigation of Stieger et al.'s [37], this protracted period allowed to confirm brain death over time and opened to the long-term pathophysiological *sequelae* of brain death adding to the clinical relevance of the model. In fact, typical cardiovascular, metabolic, and electrolyte derangements of brain death overtly occurred. A distinctive feature of this study is that we treated brain death according to standard clinical practice adopting well-accepted physiological targets [17, 18], whereas others did not [34, 36]. In fact, after brain death induction, there was cardiovascular instability, including a progressive drop of systemic vascular resistance and a rise of cardiac output that required vasoactive drugs and a positive fluid balance for normalization. To optimize ventilatory management, a lung-protective ventilatory bundle of treatment was adopted comprehensive of high PEEP - low tidal volume, apnea test execution during continuous positive airway pressure - CPAP, and lung recruitment maneuvers. As shown by Mascia et al. [23], this strategy likely contributed to prevent lung collapse and allowed to meet standard inclusion criteria for lung donation in all animals: mean PaO_2_/FiO_2_ at PEEP 5 cmH_2_O and FiO_2_ 100% was 480 ± 31 mmHg, well above the conventional threshold for acceptability set at 300 mmHg [2, 38]. Overall, management of pigs after brain death closely mimicked the clinical challenge of treating the multi-organ donors, thus offering a standardized point of reference.

While previous research has generally focused attention on either the donor or the recipient side, we have accurately reproduced the entire process of organ donation and transplantation. After brain death induction and donor treatment, the lungs were retrieved and cold-stored for 8 h. This time interval represents a realistic frame for potential lung preservation and/or reconditioning strategies. Similar to other investigations, we have then used a single lung transplantation procedure [39, 40]. Lung function early after reperfusion has been carefully monitored, as reperfusion injury is generally considered an outcome measure in lung preservation studies [41]. For this reason, based on the known effects of perfusion and ventilation on ischemia-reperfusion lung injury [42, 43], meticulous attention was given to the reperfusion protocol. The clamp on pulmonary artery was opened stepwise to allow progressive accommodation of blood flow in the newly reperfused lung vasculature and to limit as much as possible shear stress forces. This procedure also contributed to dilute residual unflushed organic acid accumulated within the lung during warm ischemia, possibly dumping the systemic effects of reperfusion. During the first minutes of reperfusion, the ventilatory component of reperfusion injury was completely abolished. Ventilation was in fact resumed only 15 min after reperfusion. At this time, attention was given to avoid ventilator-induced lung injury: a low volume - high PEEP strategy was adopted, recruitment maneuver was postponed, and residual atelectasis initially tolerated to avoid stress load to the endothelial-epithelial barrier likely to occur at high end-inspiratory lung volumes. While tolerating relative hypoxia, oxygen inspiratory fraction was kept in the low range to avoid oxidative stress [44]. These targets were only transiently set, and a full open lung strategy was resumed within 45 min. We adopted this strategy based on the opinion that a slow transition from ischemia to reperfusion is of primary importance to modulate both endothelial and epithelial ischemia-reperfusion injury in the lung.

Careful titration of pre-load indexes and ventilation settings was also adopted during post-reperfusion follow-up. This allowed to terminate the experiments with the implanted lung free of edema, as assessed by wet-to-dry lung ratio, and with normal extravascular lung water and oxygenation indexes. While other assessed the function of the implanted lung by blood gas analysis after ligation of the contralateral pulmonary artery, we implemented a different strategy. Indeed, the stress test given by the entire cardiac output flowing through a recently ischemic lung is certainly useful to reveal a possible frailty of the implanted lung. However, such evaluation protocol imposes an innatural hemodynamic challenge to both pulmonary vasculature and right heart that often leads to severe hemodynamic failure. There are in fact authors that report death at reperfusion, early after contralateral pulmonary artery ligation [45–47]. We believe that selective lung arterial and venous blood gas analysis accurately assess lung function without hemodynamic confounding factors. A limitation of this approach is that measures of respiratory mechanics reflect both native lung and graft. However, avoidance of the stress hemodynamic test allowed to better investigate the biology of ischemia-reperfusion lung injury.

In this perspective, it is interesting to note that even if all efforts to gently treat the lungs were adopted and physiological endpoints were successfully pursued during donor treatment and after transplantation, there was a clear activation of inflammation in the lung [48, 49]. In a clinical setting, achievement of these physiological parameters at the end of donor or recipient treatment would be certainly satisfactory. However, in spite of this excellent clinical outcome, enhanced expression of the chemokines CCL2-MCP-1 and IL-8 as well as increased transcription of SELE at different phases of the transplantation process suggest the presence of an inflammatory reaction [50]. Of particular interest, increased expression of IL-8 occurred during donor management. This observation confirms the idea that brain death per se induces inflammation in peripheral organs [51, 52]. Indeed, increased production of the neutrophil chemoattractant IL-8 after brain death can facilitate subsequent reperfusion injury. In addition, increased chemokine production at the Graft point can promote rejection. Of note, our reperfusion strategy protected against oxidative injury, as suggested by HMGB-1 downregulation [53, 54].

Mascia et al. showed that a conventional strategy of lung management during brain death was associated with a rise of plasma cytokines over time, while a protective strategy was not [23]. Here, we show that a lung-protective ventilation throughout brain death donor management and transplantation did not prevent the activation of inflammation in the lung. Both findings underscore the complexity of the interaction between ischemia-reperfusion and mechanical ventilation in lung transplantation and call to the need for reliable models that reproduce each phase of lung donation and transplantation. In fact, an increasing number of transplantations are performed with lungs from marginal donors, and the complex clinical settings often preclude full understanding of new treatment modalities cause-effect relationships.

There are some limitations to the study. In fact, the model resembles that of a progressively expanding mass that brings to complete ischemia, but only under the specific limitations of an animal model. The model is reproducible and with low variablity, yet some differences between animals are present (see atrial fibrillation that occurred in three animals, for instance). The animals investigated are enough to conclude about the validity of the model, but we realize that the absolute number is low, with some reflections to statistical results.

## Conclusions

In conclusion, we have set a pig model that closely resembles the entire process of organ donation and lung transplantation, and we have shown that activation of inflammation in the lung was present despite of an optimized ventilatory management throughout the protocol. The findings of our investigation may represent a starting point for various studies of lung transplantation in a standardized setting.

## Electronic supplementary material

Additional file 1: **Supplement.** A more detailed description of the section ‘Methods’ of this experimental work may be found in this Additional file. (DOC 54 KB)

Additional file 2: Figure S1: Representative diagram of the site of brain catheters placement. (TIF 65 KB)

Additional file 3: Figure S2: Radiograph of the epidural Foley catheter once inflated in the cranium of the pig. (TIF 542 KB)

Additional file 4: Figure S3: Photograph showing brain to inflated balloon proportions. (TIF 612 KB)

Additional file 5: Figure S4: Representative pattern of electroencephalogic activity before (upper panel) and after brain death induction (lower panel). (TIF 192 KB)

Additional file 6: Table S1: Controlled reperfusion parameters. (DOC 47 KB)

## Abbreviations

BD: the end of donor management after brain death
BUN: blood urea nitrogen
CCL2-MCP-1: chemokine CC motif ligand-2
CXCL-10: chemokine CXC motif ligand-10
CPP: cerebral perfusion pressure
CT: computed tomography
CVP: central venous pressure
EDN-1: endothelin-1
EELV: end-expiratory lung volume
EVLW: extravascular lung water
EtCO2: end tidal carbon dioxide
FiO2: inspiratory fraction of oxygen
GEDV: global end diastolic volume
HMGB-1: high mobility group box-1
IC: intracranial compliance
ICAM-1: intercellular adhesion molecule-1
ICP: intracranial pressure
IL-1β: interleukin-1 beta
IL-6: interleukin-6
IFNγ: interferon gamma
IL-8: interleukin-8
MAP: mean arterial pressure
OI: oxygenation index
PaO2: partial arterial pressure of oxygen
pCO2: partial pressure of carbon dioxide
PCR: polymerase chain reaction
PEEP: positive end-expiratory pressure
SpO2: peripheral saturation of hemoglobin with oxygen
SELE: selectin-E
SVV: stroke volume variation
TNFα: tumor necrosis factor alpha
VCAM-1: vascular cell adhesion molecule-1
VD/VT: dead volume to tidal volume ratio - physiological dead space
W/D: wet-to-dry ratio.
