# Supplementary material for: A standardized model of brain death, donor treatment, and lung transplantation for studies on organ preservation and reconditioning
Source: Intensive Care Med Exp. 2014 Jun 10;2:12. doi: 10.1186/2197-425X-2-12 (PMC4513016; doi:10.1186/2197-425X-2-12)
Supplement: Supplementary file 1 — Additional file 1: Supplement. A more detailed description of the section ‘Methods’ of this experimental work may be found in this Additional file. (DOC 54 KB) [file 40635_2013_14_MOESM1_ESM.doc]

# Supplements

## Anesthesia and surgical preparation of the animals

Animals received an intramuscular injection of zolanzapine and tiletamine 2 mg (Zoletil, VIRBAC Srl Milan, Italy) and medetomidine 1 mg (Dormitor, Pfizer Animal Health, Exton , PA 19341, USA, Div. of Pfizer Inc, NY, NY 10017). A continuous intravenous infusion of propofol (Diprivan, [AstraZeneca](http://en.wikipedia.org/wiki/AstraZeneca), Basiglio, Milan, Italy) 10-15 mg/Kg/h and medotomidine 3-6 γ/Kg/h was started, analgesia was assured by ketorolac 30 mg and tramadol 100 mg.

Under local anesthesia (lidocaine 2% 10 ml) a tracheostomy was performed and a 7.0 endotracheal tube Ø 7 mm was secured in place. Thereafter, a priming dose of pancuronium-bromide (6 mg) was given intravenously, followed by a continuous infusion at a rate of 0.25 mg/Kg/h. Once muscle paralysis was accomplished, mechanical ventilation was started, setting the ventilator (Datex-Ohmeda Inc., General Electrics, Madison, WI, USA) with tidal volume of 8 mL/Kg, respiratory rate according to expired CO_2_, Positive End Expiratory Pressure (PEEP) of 5 cmH_2_O and inspiratory fraction of oxygen (FiO_2_) 40%.

The right carotid artery, right internal jugular vein and left internal jugular vein were then surgically prepared and arterial (Prodimed, Plastimed Division, Saint-Leu-La-Foret, Cedex, France), central venous (7 French Arrow, Teleflex Medical, Afthlone, Ireland) and pulmonary arterial catheters (7 French Edwards Lifesciences® LLC One Edwards Way Irvine, Canada 92614) inserted and tied in place and connected to a monitoring system (Siemens® SC 8000 MDLC-1503). A dedicated 5 French catheter was placed in the right femoral artery and connected to a PiCCO® monitor (PULSION Medical Systems, AG, Stahlgruberring 28, D-81829 München, Deutschland). A Foley catheter (12 Ch Willy Rush AG, Kernen; Germany) was placed into the bladder via median skin and bladder incision. A Smart Cath catheter (Viasys, USA) was placed in the lower third of the esophagus and used to measure esophageal pressure (Pes). Continuous monitoring of temperature was obtained by a rectal probe and by the pulmonary artery catheter thermistor (Vigilance, 2012 Edwards Lifesciences® LLC, One Edwards Way, Irvine, Canada 92614).

During the first hour of surgery, 5% dextrose was infused at a rate of 30 mL/Kg, followed by a continuous infusion of balanced solutions (polisaline and normal saline) at 4-10 mL/Kg/hr. Antibiotic and antithrombotic prophylaxis were accomplished administering 1000 mg of cefazolin (Cefamezin, Pfizer Manufacturing Belgium N.V., Puurs, Belgium - endovenous) and 0,2 mL of nadroparina calcica (3800 UI, Seleparina, Italfarmaco S.p.A. – subcutaneous 0.4 mL), respectively. A dose of 500 mg methylprednisolone (Solu-medrol, Pfizer Manufacturing Belgium N.V., Puurs, Belgium - endovenous) was also administered.

After surgical preparation, the donor pigs received an isovolemic hemodilution. This was performed by drawing 1000 mL of blood from the carotid artery while continuously monitoring blood pressure and central venous pressure.

***Lung transplantation surgical procedure***

The recipient pig is placed in right lateral decubitus position and a wide left thoracotomy performed just below the tip of the scapula. The fifth rib is excised and the retractor is opened carefully to avoid bleeding from collateral branches that feed the hemi-azygos vein. The inferior pulmonary ligament is released by blunt dissection allowing subsequent preparation of the hilum. The left hemi-azygos vein and the pulmonary veins are closed and divided. The left pulmonary artery is tied and divided immediately after the first branch. A bulldog clamp is placed on the left main bronchus close to the carina. Left pneumonectomy is completed leaving a long bronchial stump. The donor lung was prepared on the back table. Division of the atrium is performed on the midline. The vein for the caudal lobe divided and closed with a 6-0 Prolene running suture to obtain an adequate atrial cuff (around the left sided pulmonary veins). The left pulmonary artery and bronchus are then divided away from the hilum and the lung brought into the pleural cavity covered by a cold lap and ice. Anastomosis are performed starting from the bronchial one, which is performed using two running sutures 5-0 Prolene C1 needle (Ethicon, Inc.). The pulmonary artery anastomosis is performed in a standard fashion using a 6-0 Prolene (Ethicon, Inc.) and left untied. Finally, the venous anastomosis is completed with a running Prolene 5-0 or 6-0 (Ethicon, Inc.). Saline is injected in the atrium immediately before tying the suture. The atrial clamp is released de-airing the donor lung in a retrograde manner, then the artery clamp is opened step by step in ten minutes; thereafter the bronchial clamp is removed allowing left lung ventilation. The thoracotomy is closed living a 20 Ch tube (Redax, S.r.l ) in the chest to drain eventual fluid.

## Respiratory mechanics

Airway pressure (Paw) and gas flow were measured at the endotracheal tube opening, while esophageal pressure (Pes) was measured from an esophageal balloon (Bicore CP-100; Irvine, CA) inflated with 0.5–1 ml of air, positioned at the lower third of the esophagus. The validity of Pes was verified by compressing the thorax with the airways occluded at end-expiration. Both flow and pressure signals were recorded on a personal computer *via* an analog-to-digital converter (Colligo; Elekton, Milan, Italy) at a sample rate of 200 Hz and stored for subsequent computer analysis. Static elastance of the total respiratory system (E,rs) was computed as Paw/VT, where Paw is the difference between plateau end-inspiratory and end-expiratory airway pressure (corrected for intrinsic PEEP) and VT is the tidal volume. Static elastance of the chest wall (E,cw) was computed as Pes/VT, where Pes is the difference between plateau end-inspiratory and end-expiratory esophageal pressure. Static lung elastance (E,l) was calculated as E,l = E,rs - E,cw.

## Lung edema

Wet to dry lung ratio (W/D ratio) was used as index of edema. Cuts of the lungs were isolated and weighted (wet weight) using a precision balance (PT 150, Sartorius AG, GØttingen, Germany). They were then heated (Swallow® fan incubator oven, LTE Scientific, Greenfield, Oldham, UK) at 50.0°C for 24 hours and weighted (dry weight). The wet to dry ratio was then calculated. W/D ratio was calculated in the right lung at the end of brain death (Brain Death) and at the end of brain death + ischemia (Ischemia); analysis was conducted on the left transplanted lung (Graft).

## RNA Isolation and Real-Time RT-PCR

Total RNA was isolated from lung base tissue biopsies by anion exchange chromatography using RNeasy Mini Kit (Qiagen Inc., Hilden, Germany). Briefly, frozen samples weighing 100–150 mg were rapidly immerged in an appropriate volume of guanidine thiocyanate buffer (Buffer RLT, Qiagen) and homogenized with Ultra-Turrax tissue homogenizer (IKA Labortechnik, Staufen, Germany). Tissue lysates were added to 70% (v/v) ethanol and immediately transferred to an RNeasy column to bind RNA to the silica-based membrane. On-colum DNase I treatment (Qiagen) was performed to remove genomic DNA contamination. After two washes with an ethanol-based buffer (Buffer RPE, Qiagen), RNA samples were eluted in RNase-free water and then quantified by optical density measurement using Nanodrop ND-100 spectrophotometer (Nanodrop Technologies, Wilmington, DE). Each sample showed a 260/280 ratio ≥ 2. RNA integrity was assessed by electrophoresis on denaturing agarose–formaldehyde gels.

Gene expression was evaluated by real-time reverse transcription polymerase chain reaction (RT-PCR) analysis based on TaqMan chemistry. Two μg of total RNA were reverse transcribed to single stranded cDNA by random priming using High-Capacity cDNA Archive Kit (Applied Biosystems, Foster City, CA, USA) according to the manufacturer's instructions. Real-time PCR was performed with 40 ng of cDNA, 1X FastStart Universal Probe Master (Roche Diagnostics GmbH, Mannheim, Germany), and predesigned 900 nM primers and 250 nM probe mix (Taqman Gene Expression Assays, Applied Biosystems), in a final volume of 12 μl. Assay IDs for target and reference genes are reported in the online supplement. Amplification reactions were carried out on an ABI PRISM 7900HT sequence detection system (Applied Biosystems) with the following thermal profile settings: an initial step of 10 minutes at 95°c to activate the FastStart Taq DNA polymerase, then 50 cycles of 95°C for 10 s and 60°C for 30 s. Three independent PCR amplification experiments were performed for each transcript. Fluorescence intensities were converted in threshold cycles (Ct) using ABI Prism SDS 2.3 software (Applied Biosystems); baseline and threshold were set by automatic analysis. Relative quantification of target gene expression was calculated with the comparative Ct method (ΔCt), using the average Ct across all basal samples as calibrator for each gene. Analysis of reference gene stability and normalization factor calculation were performed with the geNorm VBA applet version 3.4 for Excel. In the table below the gene assays used to conduct the study are shown.

| **Gene Symbol** | **Gene Name** | **Assay ID** |
| --- | --- | --- |
| ACTB | actin, beta | Ss03376081_u1 |
| GAPDH | glyceraldehyde-3-phosphate dehydrogenase | Ss03375435_u1 |
| HPRT | hypoxanthine phosphoribosyltransferase 1 | Ss03388274_m1 |
| CCL2 (MCP-1) | chemokine (C-C motif) ligand 2 | Ss03394377_m1 |
| CXCL-10 | chemokine (C-X-C motif) ligand 10 | Ss03391846_m1 |
| EDN-1 | endothelin 1 | Ss03392453_m1 |
| HMGB-1 | high mobility group box 1 | Ss03378573_u1 |
| ICAM-1 | intercellular adhesion molecule-1 | Ss03392385_m1 |
| IFN γ | interferon-gamma | Ss03391054_m1 |
| IL-1β | interleukin 1, beta | Ss03393804_m1 |
| IL-6 | interleukin 6 | Ss03384604_u1 |
| IL-8 | interleukin 8 | Ss03392435_m1 |
| SELE | selectin E | Ss03394520_m1 |
| TNF α | tumor necrosis factor | Ss03391318_g1 |
| VCAM-1 | vascular cell adhesion molecule 1 | Ss03390912_m1 |
