# Supplementary figures and images for: A standardized model of brain death, donor treatment, and lung transplantation for studies on organ preservation and reconditioning
Source: Intensive Care Med Exp. 2014 Jun 10;2:12. doi: 10.1186/2197-425X-2-12 (PMC4513016; doi:10.1186/2197-425X-2-12)

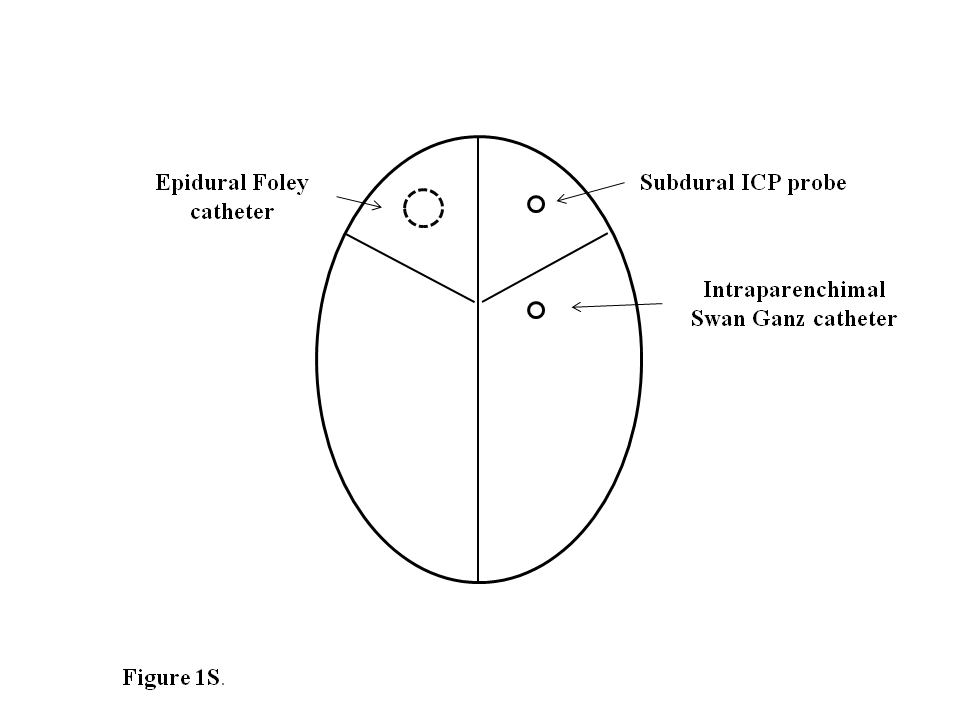

Supplement: Supplementary file 2 — Additional file 2: Figure S1: Representative diagram of the site of brain catheters placement. (TIF 65 KB) [file 40635_2013_14_MOESM2_ESM.tif]

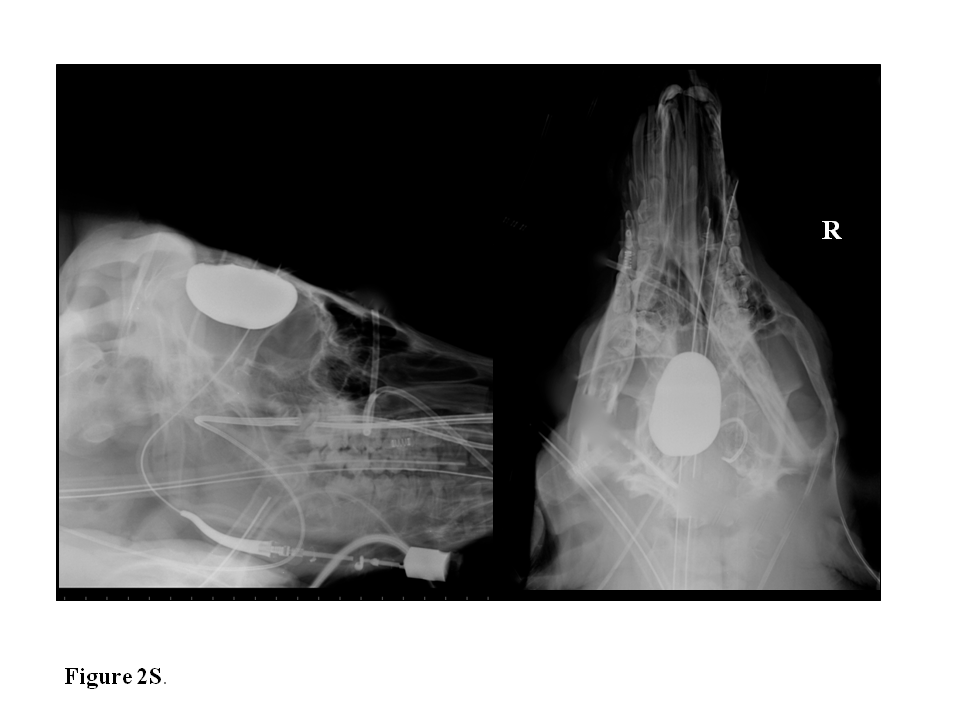

Supplement: Supplementary file 3 — Additional file 3: Figure S2: Radiograph of the epidural Foley catheter once inflated in the cranium of the pig. (TIF 542 KB) [file 40635_2013_14_MOESM3_ESM.tif]

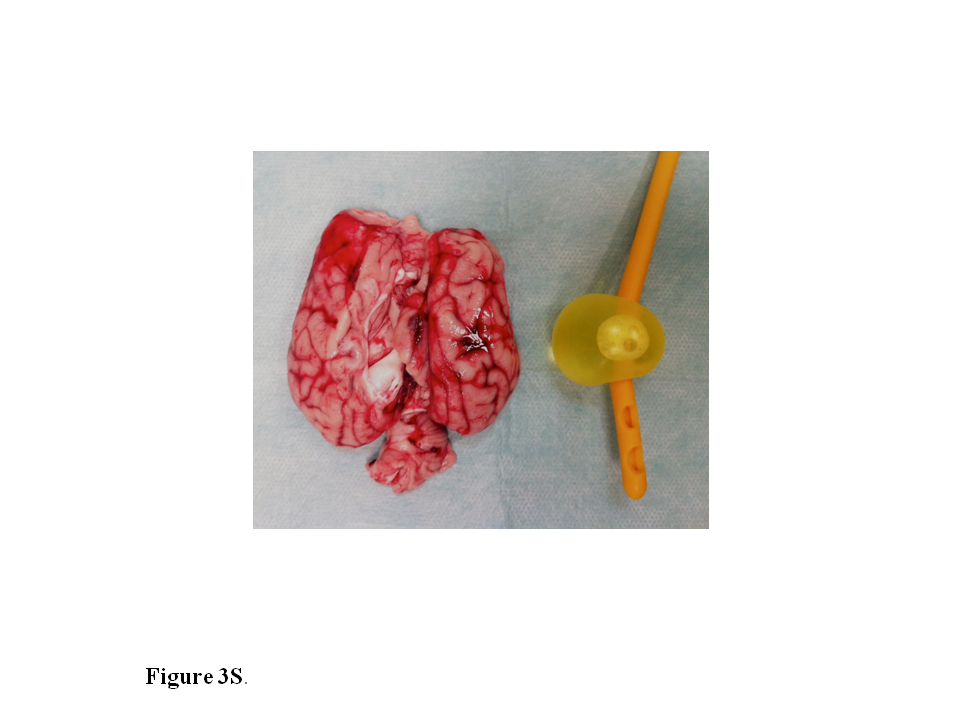

Supplement: Supplementary file 4 — Additional file 4: Figure S3: Photograph showing brain to inflated balloon proportions. (TIF 612 KB) [file 40635_2013_14_MOESM4_ESM.tif]

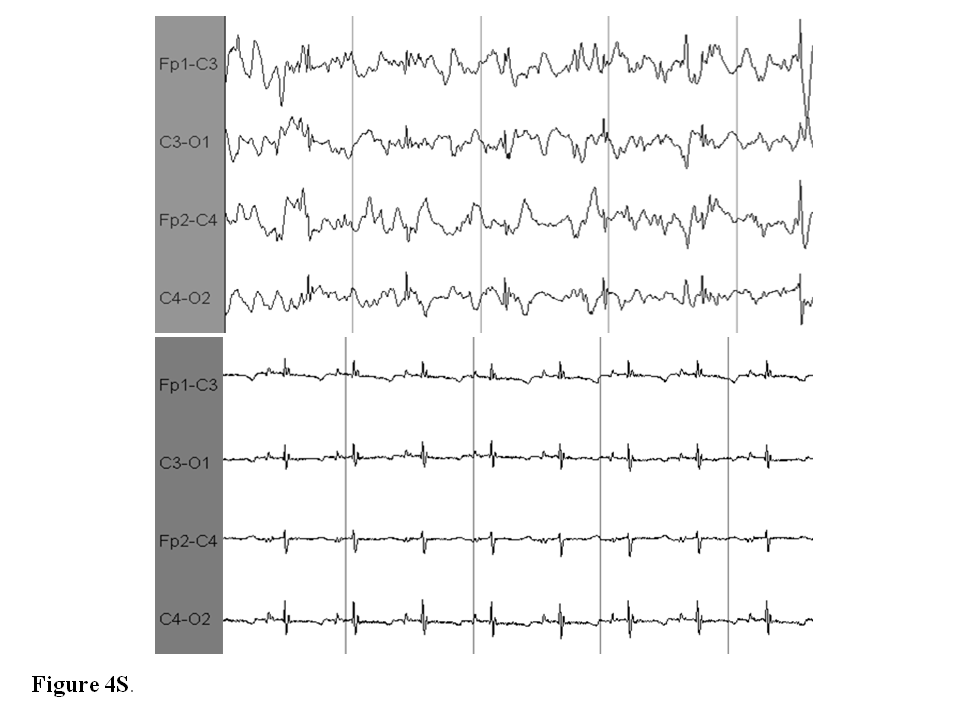

Supplement: Supplementary file 5 — Additional file 5: Figure S4: Representative pattern of electroencephalogic activity before (upper panel) and after brain death induction (lower panel). (TIF 192 KB) [file 40635_2013_14_MOESM5_ESM.tif]
