# Supplementary material for: A standardized model of brain death, donor treatment, and lung transplantation for studies on organ preservation and reconditioning
Source: Intensive Care Med Exp. 2014 Jun 10;2:12. doi: 10.1186/2197-425X-2-12 (PMC4513016; doi:10.1186/2197-425X-2-12)
Supplement: Supplementary file 6 — Additional file 6: Table S1: Controlled reperfusion parameters. (DOC 47 KB) [file 40635_2013_14_MOESM6_ESM.doc]

**Table 1S.**

**Controlled reperfusion parameters.**

Table shows respiratory and cardiovascular parameters collected during the first hour of reperfusion.

The artery clamp was opened step by step in ten minutes (0 – 10 minutes). Zero represents the beginning of the reperfusion time. After 15 minutes from the start of the controlled reperfusion the bronchial clamp was removed allowing left lung ventilation. A recruitment maneuver was performed forty five minutes after the start of controlled reperfusion. OLV: One Lung Ventilation; BI: Bi-pulmonary ventilation; PEEP: positive end-expiratory pressure; PAP: mean pulmonary artery pressure; CVP: central venous pressure.

Data are presented as mean ± standard deviation. One Way ANOVA Repeated Measures. P<0.05 accepted as significant: ***** vs. 0; **^§^** vs. -10.

| **Time, min** | **-10** | **0** | **5** | **10** | **15** | **20** | **25** | **30** | **45** | **60** | **P** |
| --- | --- | --- | --- | --- | --- | --- | --- | --- | --- | --- | --- |
| Ventilatory Mode | OLV | OLV | OLV | OLV | OLV | BI | BI | BI |  | BI |  |
| Mean Airway Pressure, cmH_2_O | 9.0±1.2 | 9.0±1.4 | 10.2±1.3 | 11.4±0.9 | 11.6±1.1^§*^ | 11.2±1.1 | 11.4±1.5 | 11.4±1.6^§*^ | **Recruitment maneuver** | 12.0±1.6 | <0.05 |
| Peak Airway Pressure, cmH_2_O | 20.2±3.6 | 19.5±4.6 | 19.0±3.4 | 19.5±1.9 | 20.7±1.5 | 18.5±1.9 | 19.7±1.7 | 19.8±2.2 |  | 20.8±3.1 | 0.953 |
| Tidal Volume, mL/Kg | 7.1±1.4 | 6.8±1.2 | 6.7±1.6 | 6.9±1.2 | 6.4±0.7 | 6.1±0.7 | 6.0±0.5 | 6.5±0.7 |  | 7.4±1.2 | 0.100 |
| Tidal Volume, mL | 296±46 | 295±42 | 279±36 | 295±69 | 273±63 | 259±30 | 255±49 | 276±45 |  | 321±99 | 0.105 |
| PEEP, cmH_2_O | 6.0±1.2 | 6.2±1.9 | 7.4±1.3 | 8.6±0.9 | 8.6±0.9 | 8.4±0.9 | 8.2±1.1 | 8.2±1.1 |  | 8.2±1.1 | <0.05 |
| End tidal CO_2_, mmHg | 53±4 | 55±7 | 59±9 | 59±11 | 62±8 | 63±5 | 65±5^§^ | 63±3 |  | 57±4 | <0.05 |
| Respiratory Rate, breaths/min | 20±4 | 18±4 | 18±4 | 17±3 | 18±3 | 19±2 | 19±2 | 19±2 |  | 19±2 | 0.953 |
| FiO_2_, % | 40 | 40 | 40 | 40 | 40 | 40 | 40 | 40 |  | 40 | 1 |
| Temperature, °C | 38.1±0.6 | 37.7±0.5 | 37.9±0.6 | 37.9±0.9 | 37.8±0.7 | 37.9±0.7 | 37.9±0.7 | 37.9±0.7 |  | 37.9±1 | 0.933 |
| Heart Rate, beats/min | 103±21 | 103±24 | 102±23 | 101±21 | 103±21 | 100±22 | 99±24 | 103±33 |  | 102±27 | 0.220 |
| MAP mmHg | 93±19 | 106±18 | 87±10 | 81±6 | 100±16 | 95±24 | 86±21 | 92±18 |  | 96±21 | 0.178 |
| PAP, mmHg | 25.6±5.6 | 24.8±4.3 | 25.4±4.5 | 25.6±5.3 | 27.2±4.6 | 26.2±4.9 | 26.2±4.4 | 25.8±4.8 |  | 24.6±2.9 | 0.433 |
| CVP, mmHg | 6.4±1.7 | 6.4±1.7 | 6.6±2.3 | 6.8±1.8 | 7.2±2.3 | 7.0±2.0 | 7.0±1.9 | 7.0±1.9 |  | 7.5±2.5 | 0.416 |
